# In-Cell RNA Hydrolysis Assay: A Method for the Determination of the RNase Activity of Potential RNases

**DOI:** 10.1007/s12033-015-9844-7

**Published:** 2015-01-30

**Authors:** Youngsil Seo, Hye-Ryeong Jun, Joungmin Lee, Hyunjoon Park, Minjae Kim, Youngsoo Lee, Myung-Hee Kwon

**Affiliations:** 1 Department of Biomedical Sciences, Graduate School, Ajou University, 206 World Cup-ro, Yeongtong-gu, Suwon, 443-749 South Korea; 2 Department of Microbiology, Ajou University School of Medicine, 206 World Cup-ro, Yeongtong-gu, Suwon, 443-749 South Korea; 3 Genomic Instability Research Center, Ajou University School of Medicine, 206 World Cup-ro, Yeongtong-gu, Suwon, 443-749 South Korea

**Keywords:** RNA, RNase, Hydrolysis, Catalytic antibody, Assay, In-cell

## Abstract

Conventional procedures to assay RNA degradation by a protein with ribonuclease (RNase) activity require a step to isolate intact RNA molecules, which are used as a substrate. Here, we established a novel “In-cell RNA hydrolysis assay” in which RNAs within cells are used as a substrate for the RNA-hydrolyzing protein, thereby avoiding the need to prepare intact RNA molecules. In this method, the degree of RNA degradation is indicated by the fluorescence intensity of RNA molecules released from fixed and permeabilized cells following treatment with the potential RNase. A catalytic 3D8 antibody capable of degrading RNAs and pancreatic RNase A were used as model RNases. Our data demonstrate that the novel In-cell RNA hydrolysis assay is a reliable and sensitive method to analyze the activities of potential RNA-hydrolyzing proteins such as catalytic antibodies.

## Introduction

Some anti-DNA antibodies that are found at high titers in humans and mice with autoimmune diseases, such as systemic lupus erythematosus and multiple sclerosis [1, 2], are catalytic antibodies, also called abzymes, capable of hydrolyzing DNA or RNA polymers [3–5]. It was reported that polyclonal antibodies against DNA or RNA can hydrolyze both molecules [6, 7]; however, it is unclear whether these findings are due to the activity of a single antibody that can hydrolyze both DNA and RNA or to the activities of several antibodies that can each hydrolyze DNA or RNA. Therefore, analysis of the RNA-hydrolyzing activity of catalytic monoclonal anti-DNA antibodies (more appropriately, catalytic anti-nucleic acid antibodies) is important to understand the properties of these antibodies.

Several methods have been developed to analyze the DNA-hydrolyzing activity of catalytic anti-DNA antibodies, in which plasmid DNA or synthetic nucleotides are used as a substrate [8–12]. These methods can be used to analyze the RNA-hydrolyzing capacity of proteins with RNase activity, such as anti-DNA antibodies, simply by substituting the DNA substrates (plasmid DNA or synthetic deoxyribonucleotides) for RNA substrates (ribosomal RNA (rRNA) or synthetic ribonucleotides). We have previously used in situ gel electrophoresis [13] and fluorescence resonance energy transfer-based analysis [14] to assay the RNA-hydrolyzing activity of a catalytic 3D8 single-chain variable fragment (scFv) antibody with DNA- and RNA-hydrolyzing capacities. However, RNA molecules are extremely vulnerable to degradation by RNases, and RNA samples are often contaminated with these enzymes during their preparation and storage, resulting in high background in RNA hydrolysis assays.

Here, we developed an “In-cell RNA hydrolysis assay” in which RNA molecules within cells are used as a substrate, thereby avoiding the need to isolate intact RNA from cells or to synthesize RNA. Two proteins with RNase activity, 3D8 scFv antibody and ribonuclease (RNase) A, were used as model proteins to test this application. In this assay, degradation of cellular RNAs is simply indicated by the fluorescence intensity of RiboGreen-labeled RNA molecules released from fixed and permeabilized cells upon treatment with the RNase. This was verified by the finding that the amount of RNAs inside fixed and permeabilized cells was decreased after treatment with an RNase. The stringency of this method was confirmed by the finding that cellular DNA did not affect the fluorescence intensity of RiboGreen-labeled RNA. This assay provides a simple means of determining the RNase activity of potential RNases including catalytic antibodies.

## Materials and Methods

### Preparation of scFv Proteins

3D8 scFv and HW6 scFv proteins containing a protein-A tag were purified from the supernatants of bacterial cultures using IgG-Sepharose affinity chromatography [8].

### Analysis of the Fluorescence Intensity of RiboGreen-Labeled RNA Fragments

To detect cellular RNA fragments released from cells using RiboGreen reagent, A549 human lung epithelial cells grown at a density of 5 × 10^4^ cells/well in a 48-well plate were washed three times with ice-cold PBS and fixed with 4 % paraformaldehyde prepared in PBS for 15 min at room temperature (RT). Cells were washed twice with PBS, and cell membranes were permeabilized with *P* buffer (1 % bovine serum albumin, 0.1 % saponin, and 0.1 % sodium azide prepared in PBS) for 30 min at RT. Thereafter, cells were treated with 100 μl of various concentrations of 3D8 scFv antibody, HW6 scFv antibody, or RNase A (Intron Biotech) prepared in PBS containing 1 mM MgCl_2_ (pH 7.2) for 2 h at 37 °C. Next, 50 μl of the conditioned medium was added to microplate wells containing 100 μl of Quant-iT™ RiboGreen^®^ reagent (Invitrogen, Cat. No. R11490) diluted 200-fold with TE buffer (10 mM Tris–HCl and 1 mM EDTA, pH 7.5) and incubated for 2 min at RT in the dark. The reaction mixtures were excited at 485 nm and emission at 530 nm was measured using a fluorescence analyzer.

In some cases, conditioned medium of fixed and permeabilized cells, pure 16S and 23S rRNA from *E. Coli* (Invitrogen), or 1 μg/ml pUC19 plasmid DNA was incubated with RiboGreen reagent in the absence or presence of 2 U DNase I (NEB) prepared in PBS containing 1 mM MgCl_2_ (pH 7.2) or in DNase I reaction buffer (10 mM Tris–HCl, 2.5 mM MgCl_2_, and 0.5 mM CaCl_2_, pH 7.6).

### Analysis of RNA Fragments by Absorbance at 260 nm (A_260_)

To detect cellular RNA fragments released from cells by measuring ultraviolet (UV) light absorbance, A549 cells grown at a density of 5 × 10^5^ cells/well in a 6-well plate were fixed and permeabilized as described above. Thereafter, cells were treated with 500 μl of 10 μM RNase A or 3D8 scFv antibody prepared in PBS containing 1 mM MgCl_2_ (pH 7.2) for 2 h at 37 °C. Then, 150 μl of conditioned medium from each well was applied to a protein precipitation kit (National Diagnostics, Cat. No. EC-888) to remove protein contaminants, and A_260_ was measured using a UV spectrophotometer.

### Confocal Microscopy

To analyze the level of RNA inside cells, confocal microscopy was performed using a Click-iT^®^ RNA Alexa Fluor^®^ 488 Imaging Kit (Molecular Probes). A549 cells grown on coverslips (5 × 10^4^ cells/well in a 24-well plate) were incubated with 1 mM 5-ethynyl uridine (EU) for 20 h at 37 °C, allowing the incorporation of EU into newly synthesized RNAs. Cells were fixed and permeabilized as described above and then treated with 500 μl of 10 μM RNase A or 3D8 scFv antibody prepared in PBS containing 1 mM MgCl_2_ (pH 7.2) for 2 h at 37 °C. Cells were incubated with Alexa 488-azide solution, which ligates EU, for 30 min at RT in the dark, and washed three times with RNase-free PBS. Nuclei were stained with Hoechst 33342 (Vector Laboratories) for 30 min at RT. Images of intracellular green fluorescence were acquired by confocal microscopy (Carl Zeiss LSM 710).

### Flow Cytometry

To detect cells containing fluorescent RNA molecules, A549 cells (5 × 10^5^ cells/well in a 6-well plate) were incubated with 1 mM EU for 20 h at 37 °C and then detached by trypsin treatment. Cells were washed, fixed, and permeabilized as described for confocal microscopy experiments. After treatment with 800 μl of 10 μM RNase A or 3D8 scFv antibody prepared in PBS containing 1 mM MgCl_2_ (pH 7.2) for 2 h at 37 °C, cells were incubated with Alexa 488-azide solution, which ligates EU, for 30 min at RT and washed three times with RNase-free PBS. Finally, cells were suspended in 4 % paraformaldehyde prepared in PBS, and intracellular green RNA fluorescence was analyzed using a FACSCanto II flow cytometer (Becton–Dickinson).

## Results

### RNA Degradation by a Potential RNase can be Monitored Using the In-Cell RNA Hydrolysis Assay

**Fig. 1:**
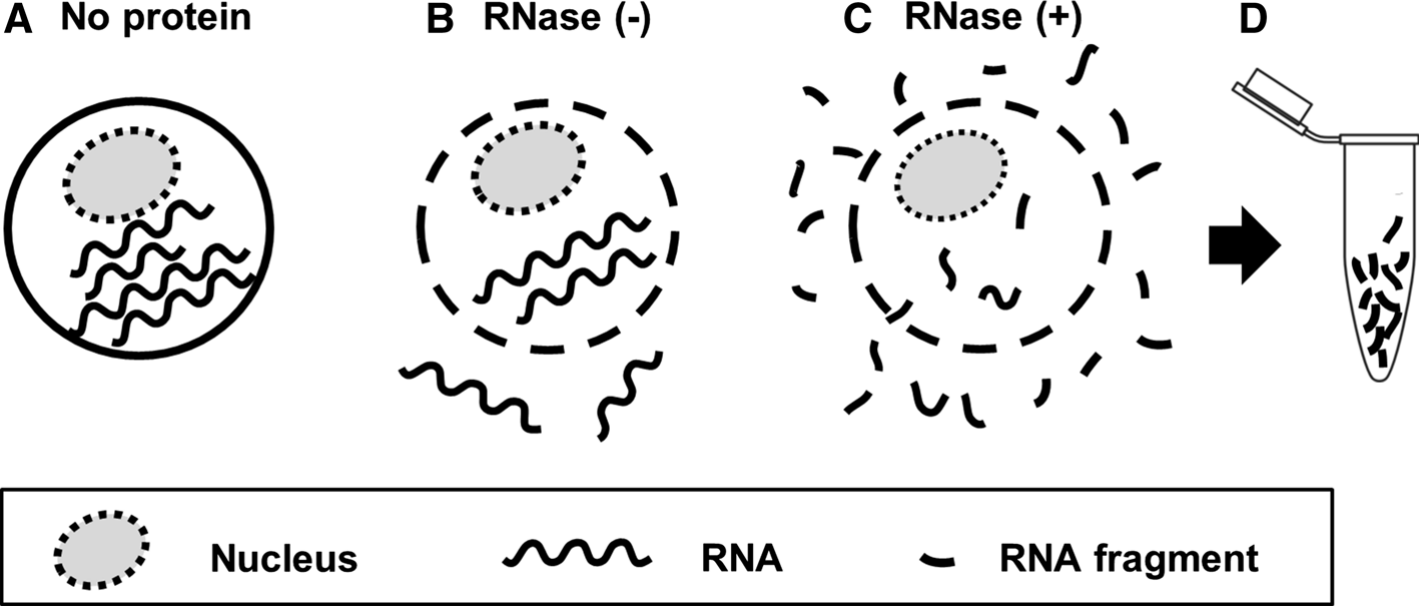
Schematic representation of the concept of the In-cell RNA hydrolysis assay. **a** Cells containing intact RNAs. **b** Cells whose membranes have been permeabilized, allowing the release of small RNA fragments even in the absence of RNase treatment. **c** Cells release small RNA fragments via their membrane pores following treatment with an RNase. **d** Collection of degraded RNA fragments released from cells, which are labeled with RiboGreen and subjected to fluorescence intensity measurement

**Fig. 2:**
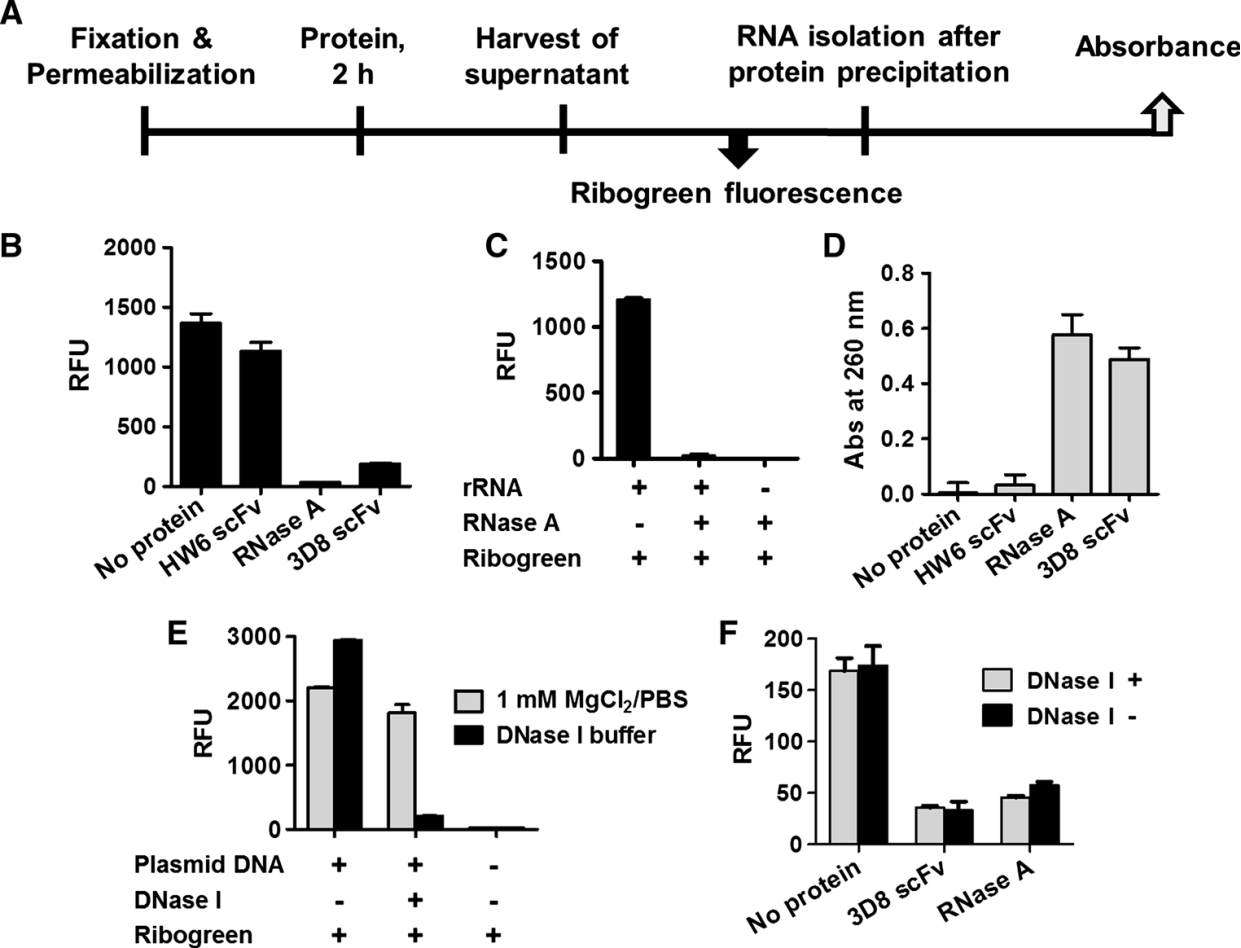
The detection of RNAs released from fixed and permeabilized cells. **a** Experimental procedures for the data presented in (**b**) and (**d**). **b** A549 cells grown in a 48-well plate at a density of 5 × 10^4^ cells/well were fixed, permeabilized, and incubated with 100 μl of 10 μM RNase A or 10 μM 3D8 antibody for 2 h at 37 °C. An aliquot of the conditioned medium was added to RiboGreen, and the fluorescence intensity was analyzed. **c** Pure 16S and 23S rRNA from *E. coli* was incubated with RiboGreen in the presence or absence of RNase A prior to fluorescence intensity analysis. **d** A549 cells grown in a 6-well plate at a density of 5 × 10^5^ cells/well were fixed, permeabilized, and incubated with 10 μM RNase A or 3D8 antibody for 2 h at 37 °C. Proteins were removed from the conditioned medium by precipitation, and absorbance at 260 nm was measured. **e** Degradation of plasmid DNA (1 μg/ml) by DNase I (2 U) was tested using RiboGreen prepared in DNase I reaction buffer for 2 h at 37 °C. **f** Fixed and permeabilized cells in a 48-well plate were treated with RNase A or 3D8 antibody (10 μM) in the presence or absence of DNase I (2 U) for 2 h at 37 °C. The conditioned medium was mixed with RiboGreen prior to fluorescence intensity analysis. *RFU* relative fluorescence unit. Data represent the mean ± standard error of four independent experiments

When developing the novel In-cell RNA hydrolysis assay using RiboGreen, we postulated that if cellular RNAs are degraded by potential RNases in fixed and permeabilized cells, cleaved small RNA fragments would be released from the cells and could be detected using RiboGreen reagent (Fig. 1). Fixed and permeabilized cells were incubated with 3D8 scFv antibody, HW6 scFv antibody, or RNase A, and then the level of RNA in the conditioned medium was assessed in two ways (Fig. 2a): the fluorescence intensity of RiboGreen and A_260_.

RiboGreen binds to base-pairing nucleic acids and emits green fluorescence signals, whereas free RiboGreen exhibits negligible fluorescence in solution [15]. When mixed with RiboGreen reagent, fluorescence signals were not detected in the conditioned medium of cells treated with RNase A or 3D8 scFv antibody, which can hydrolyze RNA (Fig. 2b). Fluorescence signals were detected when RiboGreen was mixed with intact rRNA, but not when it was mixed with rRNA that had been completely degraded by RNase A (Fig. 2c). Based on this finding, we speculated that complete degradation of RNA by RNase A or 3D8 scFv antibody can perturb the formation of intrastrand base-pairs to which RiboGreen binds, resulting in an undetectable fluorescence signal. Strong fluorescence was observed in negative controls (untreated cells and cells treated with HW6 scFv antibody, which does not cleave RNA) (Fig. 2b). Such fluorescence signals in negative control samples suggest that some RNA molecules are small enough to be released from permeabilized cells even in the absence of an RNase.

Direct evidence for the release of small RNA fragments upon degradation by RNase A or 3D8 scFv antibody is supported by the measurement of A_260_ in conditioned medium. A_260_ is proportional to the total amount of RNA or bases, regardless of the integrity of the RNA molecules. The A_260_ of conditioned medium from which proteins had been removed using a protein precipitation kit was inversely proportional to the fluorescence intensity of RiboGreen in the corresponding samples (Fig. 2d). These A_260_ values were significantly increased when fixed and permeabilized cells were treated with RNase A or 3D8 scFv antibody, suggesting that RNAs are easily released from these cells when they are degraded by RNases. A_260_ was negligible in untreated and HW6 scFv antibody-treated samples, which is inconsistent with the strong signal shown in Fig. 2b. This can be explained by differences in assay sensitivity; the RiboGreen^®^ assay is ~1,000-fold more sensitive than A_260_ measurements. The sensitivities of the RiboGreen and A_260_ assays are approximately 1 ng/ml and 4 μg/ml RNA in solution, respectively, according to the manufacturer’s guide.

To improve the reliability of the In-cell RNA hydrolysis assay using RiboGreen, it was necessary to rule out the possibility that DNA molecules interfere with the results because RiboGreen can bind single-stranded and double-stranded DNA, in addition to RNA. The effect of DNA on RiboGreen fluorescence was examined in DNase I reaction buffer because DNase I works in this buffer containing CaCl_2_ but not in PBS containing 1 mM MgCl_2_ (Fig. 2e). The fluorescence intensity of RiboGreen in the conditioned medium of cells treated with RNase A or 3D8 scFv antibody was similar in the presence and absence of DNase I. This suggests that DNA does not affect the detection of RNA by RiboGreen in this assay (Fig. 2f). Taken together, these data show that potential RNases enter fixed and permeabilized cells and degrade RNA into fragments that are small enough to be released from cells through membrane pores, thereby allowing RNA degradation to be monitored.

### RNA is Degraded in an RNase Concentration-Dependent Manner

**Fig. 3:**
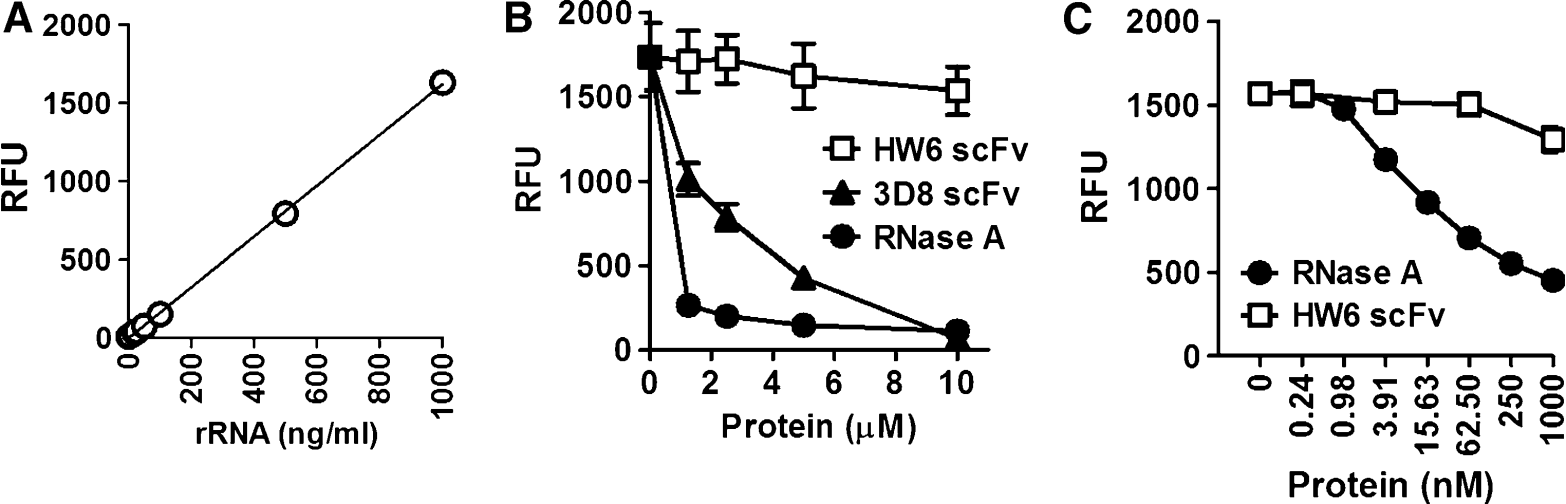
Quantitative analysis of RNA degradation in fixed and permeabilized cells treated with RNase A or 3D8 antibody. **a** Standard curve to determine the concentration of RNA. Pure rRNA at known concentrations was mixed with RiboGreen prior to fluorescence intensity analysis. **b** Dose-dependent RNA degradation. RNA degradation was quantitatively analyzed in fixed and permeabilized cells (5 × 10^4^ cells) treated with 1.25–10 μM RNase A or 3D8 antibody. **c** The sensitivity of this method. RNA degradation was quantitatively analyzed in fixed and permeabilized cells treated with various dilutions of RNase A generated from a stock solution of 1 μM. RFU, relative fluorescence unit. Data represent the mean ± standard error of four independent experiments

RNA degradation in fixed and permeabilized cells treated with various concentrations of proteins was quantitatively analyzed. A standard curve was generated using pure 16S and 23S rRNA to calculate the concentration of RNA released from fixed and permeabilized cells upon treatment with an RNase (Fig. 3a). In the absence of RNase treatment, 100 µl of conditioned medium from a single well of a 48-well plate (5 × 10^4^ cells/well) contained ~400 ng of RNA. This supports the observation that RNA released from untreated cells in a 48-well plate could not be detected by measurement of A_260_ but was detected by RiboGreen labeling (Fig. 2). The degradation of RNA molecules, as detected by RiboGreen fluorescence in conditioned medium, increased in an RNase A dose- and 3D8 scFv antibody dose-dependent manner (Fig. 3b). In total, 100 μl of 10 μM 3D8 scFv antibody, 10 μM HW6 scFv antibody, and 10 μM RNase A corresponds to 3.7, 3.7, and 1.3 μg of protein, respectively.

Furthermore, the sensitivity of our method was assessed with RNase A. RNase A was diluted to various concentrations from a stock solution of 1 µM, and then 100 µl of each dilution was incubated with fixed and permeabilized cells, prior to detection of the RiboGreen signal. RNA hydrolysis was detectable with 3.91 nM RNase A (Fig. 3c), indicating a sensitivity of ~5 ng (100 μl of 3.91 nM RNase A corresponds to 5.4 ng of protein). This method seems to be ~400-fold more sensitive than the traditional in situ gel electrophoresis-based method [13], which requires ~2 μg of protein. Therefore, it should be possible to assay the activities of RNase candidates that are lowly abundant using this assay.

### Verification of the Principle of the In-Cell RNA Hydrolysis Assay by the Reduction in the Level of Intracellular RNA Following RNase Treatment

**Fig. 4:**
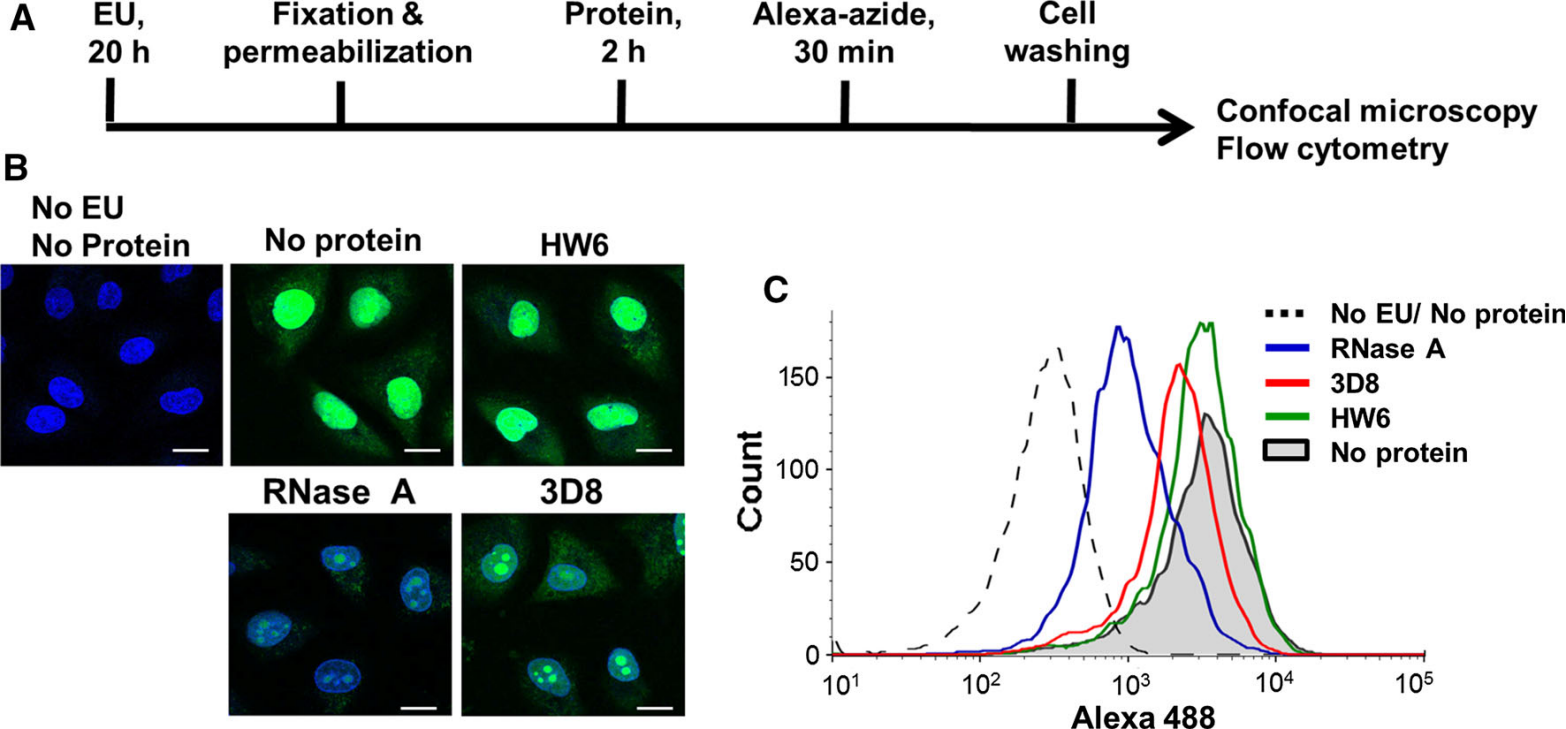
Detection of RNA in fixed and permeabilized cells treated with RNase A or 3D8 antibody. **a** Schematic diagram of the experimental procedure to detect the level of RNA in cells according to the fluorescence intensity of RiboGreen. **b**, **c** A549 cells plated in a 24-well plate (**b**) or a 6-well plate (**c**) were incubated with 5-ethynyl uridine (EU) for 20 h, fixed, permeabilized, and incubated with 10 μM RNase A or 3D8 antibody for 2 h at 37 °C. Cells were applied to click chemistry for 30 min, extensively washed, and examined by confocal microscopy (**b**) and flow cytometry (**c**). Data are representative of three independent experiments. *Scale bars* represent 10 μm in (**b**)

To verify the principle of the In-cell RNA hydrolysis assay, we examined whether the amount of RNA within cells decreased as RNA was released. Cells were incubated with EU, which is actively incorporated into nascent RNAs in the nucleus, fixed, permeabilized, and treated with RNase A or 3D8 scFv antibody. Following labeling of EU-incorporated RNAs with Alexa 488-azide, RNA remaining inside cells was analyzed by confocal microscopy (Fig. 4a) and flow cytometry (Fig. 4b). The fluorescence intensity of RNA inside fixed and permeabilized cells treated with RNase A or 3D8 scFv antibody was dramatically decreased, in comparison with levels inside negative control cells. This was indeed due to the release of degraded RNA from cells. This verifies the principle of the In-cell RNA hydrolysis assay i.e., RNA inside cells can be used as a substrate for a potential RNase.

## Discussion

We established the novel In-cell RNA hydrolysis assay. This assay was designed to detect the release of degraded RNA generated by a RNA-hydrolyzing protein from fixed and permeabilized cells using RiboGreen, an ultrasensitive reagent to RNA in solution. We expected that the fluorescence intensity of RiboGreen would be directly proportional to the amount of RNA fragments released from permeabilized and fixed cells treated with an RNase; however, the fluorescence intensity of RiboGreen was decreased by treatment with an RNase due to the complete degradation of RNA molecules such that they were no longer recognized by RiboGreen (Fig. 2b). Therefore, this assay detects the reduction in the level of intact RNA molecules in RNase-treated cells, in comparison with the basal level in untreated cells. This assay is simple and requires neither RNAs purified from cells nor artificially synthesized RNA molecules to be used as substrates for potential RNases. Moreover, it does not require a DNase treatment step to improve its reliability, even though RiboGreen can bind both RNA and DNA. The fluorescence intensity of RiboGreen was similar in the presence and absence of DNase I (Fig. 2f). DNA molecules packaged into chromatin in the nucleus would not be attacked by a protein with DNA- and RNA-hydrolyzing activities, such as 3D8 scFv antibody; therefore, the majority of RiboGreen fluorescence in this assay can be attributed to RNA, not to DNA.

Cells were fixed with 4 % paraformaldehyde and permeabilized with 0.1 % saponin. Paraformaldehyde is a protein-crosslinking fixative that creates covalent bonds between proteins, primarily residues of the basic amino acid lysine, without affecting nucleic acids. This indicates that nucleic acids can be freely released from fixed cells, while the majority of proteins would be retained [16]. Saponin generates pores on membranes by forming complexes with cholesterol in the membrane [17]. Treatment with saponin generates pores in cell membranes through which small nucleic acids can passively diffuse; however, it does not permeabilize the inner nuclear envelope and the mitochondrial membrane, which lack cholesterol. Saponin generates pores with a diameter of approximately 8 nm [18]. Given that molecules larger than 100 kD cannot pass through pores with a diameter of 10 nm [19], it seems that RNAs shorter than ~250 nt were released from fixed and permeabilized cells in the absence of RNase treatment, and these short RNAs underlie the RiboGreen fluorescence detected in negative control samples in Fig. 2.

The level of RNA in nuclei was dramatically reduced in cells treated with RNase A or 3D8 scFv antibody (Fig. 4b). This could be because the main target of these RNases is naked rRNAs in nuclei, including 45S (13.7 kb) pre-rRNA, 28S (5,070 nt) rRNA, 18S (1,869 nt) rRNA, 5.8S (156 nt) rRNA, and 5S (121 nt) rRNA, which are yet to assemble with proteins into ribosomes. rRNAs comprise approximately 80 % of total RNA in eukaryotic cells, while mRNAs and tRNAs (~75–90 nt) comprise approximately 5 and 15 %, respectively [20]. RNase A (14 kD) and 3D8 scFv antibody (34 kD) likely entered the nucleus via nuclear pores to attack rRNAs. The diameter of the nuclear pore is approximately 9 nm [19]. Therefore, RNA fragments of less than ~300 nt can be released from the nucleus, even though saponin does not affect the nuclear membrane. There was a marked basal level of small intact RNAs released from fixed, untreated cells (Fig. 2b). Although the experiment shown in Fig. 4 sought to verify the principle of the In-cell RNA hydrolysis assay, it could also be used as another method to assay RNA degradation.

Our study demonstrates that the In-cell RNA hydrolysis assay can be a useful alternative method to analyze RNase activity.

## Abbreviations

A_260_: Absorbance at 260 nm
EU: 5-Ethynyl uridine
RNase: Ribonuclease
scFv: Single-chain variable fragment
rRNA: Ribosomal RNA
RT: Room temperature
UV: Ultraviolet
